# Supplementary material for: DMRforPairs: identifying Differentially Methylated Regions between unique samples using array based methylation profiles
Source: BMC Bioinformatics. 2014 May 15;15:141. doi: 10.1186/1471-2105-15-141 (PMC4046028; doi:10.1186/1471-2105-15-141)
Supplement: Additional file 2 — DMRforPairs output for the comparison of A431-MCF7 and NA17018-NA17105. Please start from the HTML files in each folder. Available via the BMC Bioinformatics website. [file 1471-2105-15-141-S2.zip › 1394847754114233_MOESM2_ESM/A431_MCF7/figures/23097.pdf]

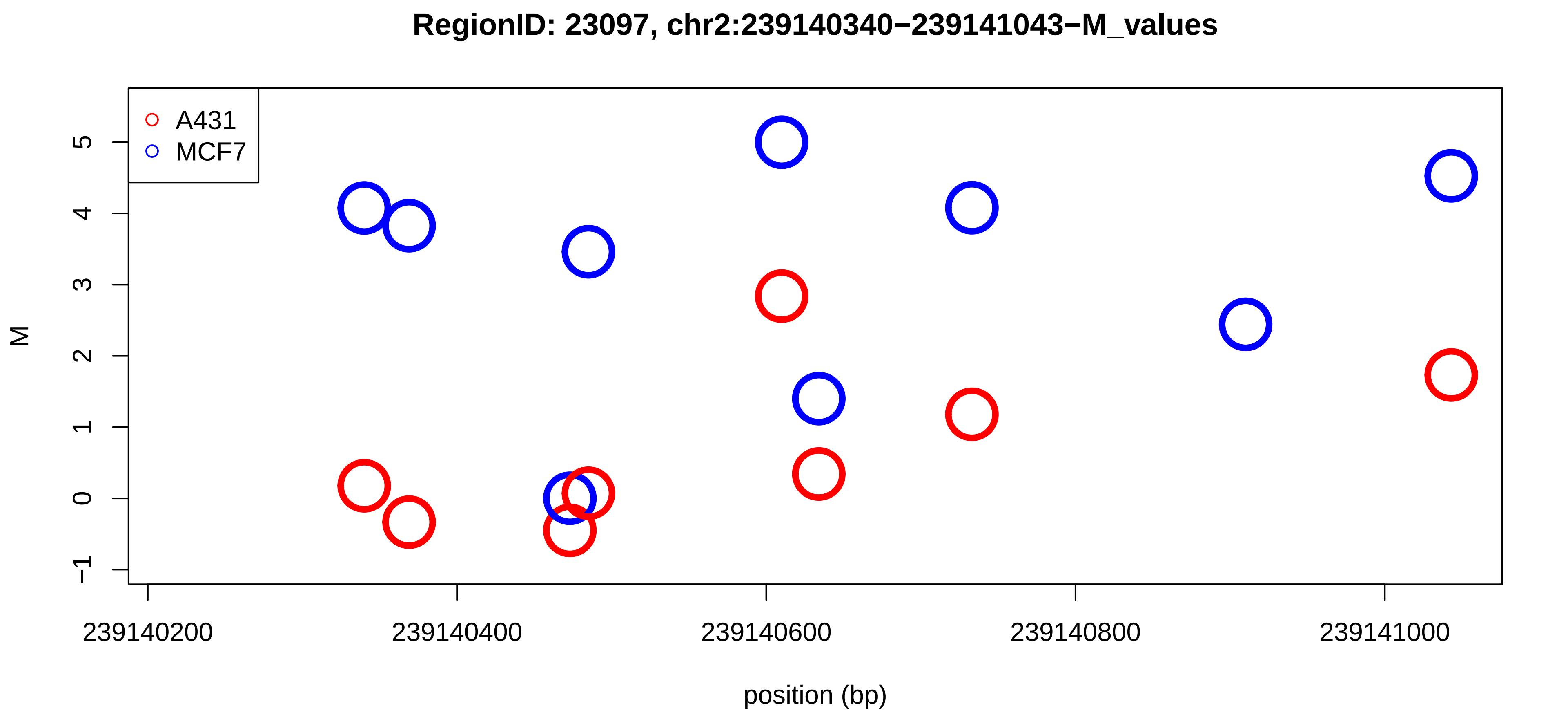
RegionID: 23097, chr2:239140340−239141043−M_values
A431
MCF7
M
5
4
3
2
1
0
−1
239140200
239140400
239140600
239140800
239141000
position (bp)

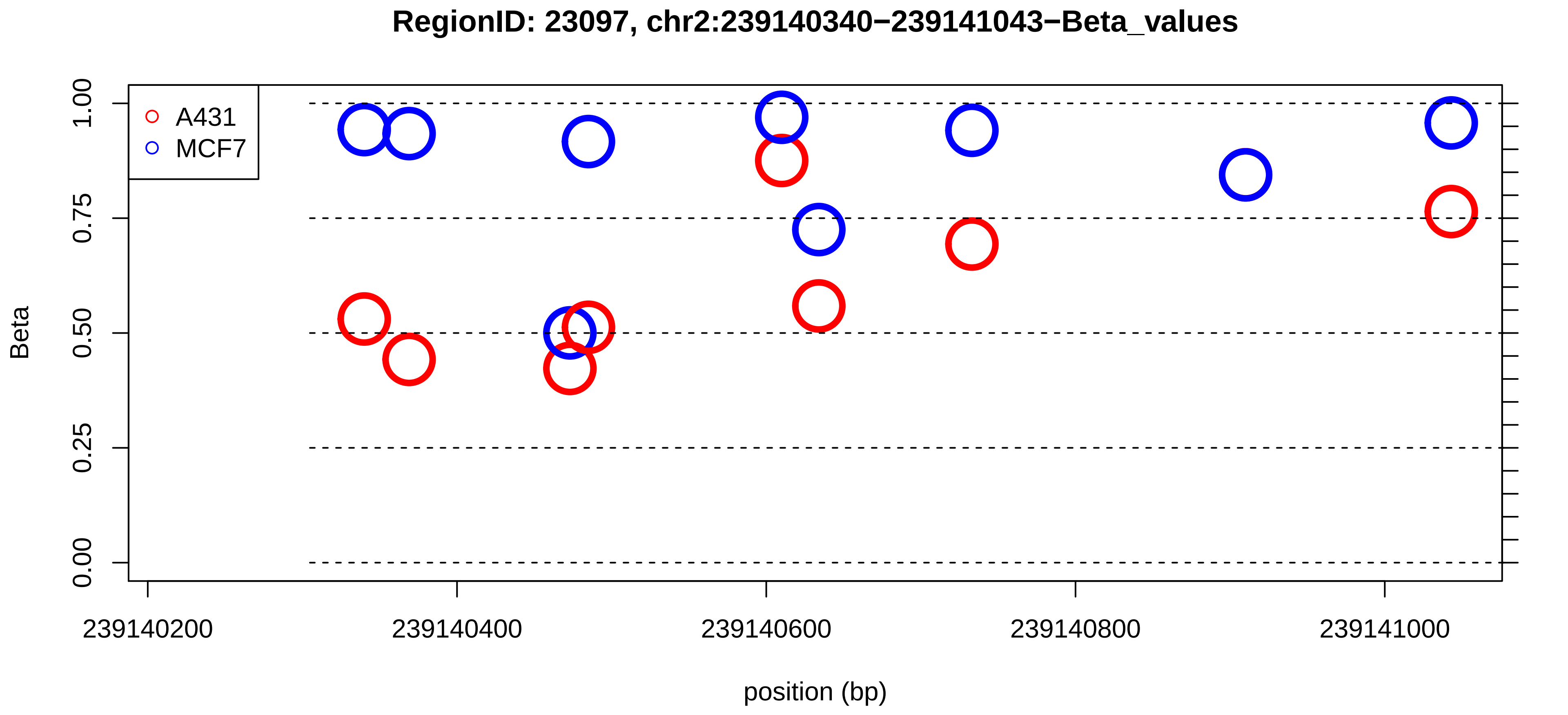
RegionID: 23097, chr2:239140340−239141043−Beta_values
A431
MCF7
Beta
1.00
0.75
0.50
0.25
0.00
239140200
239140400
239140600
239140800
239141000
position (bp)

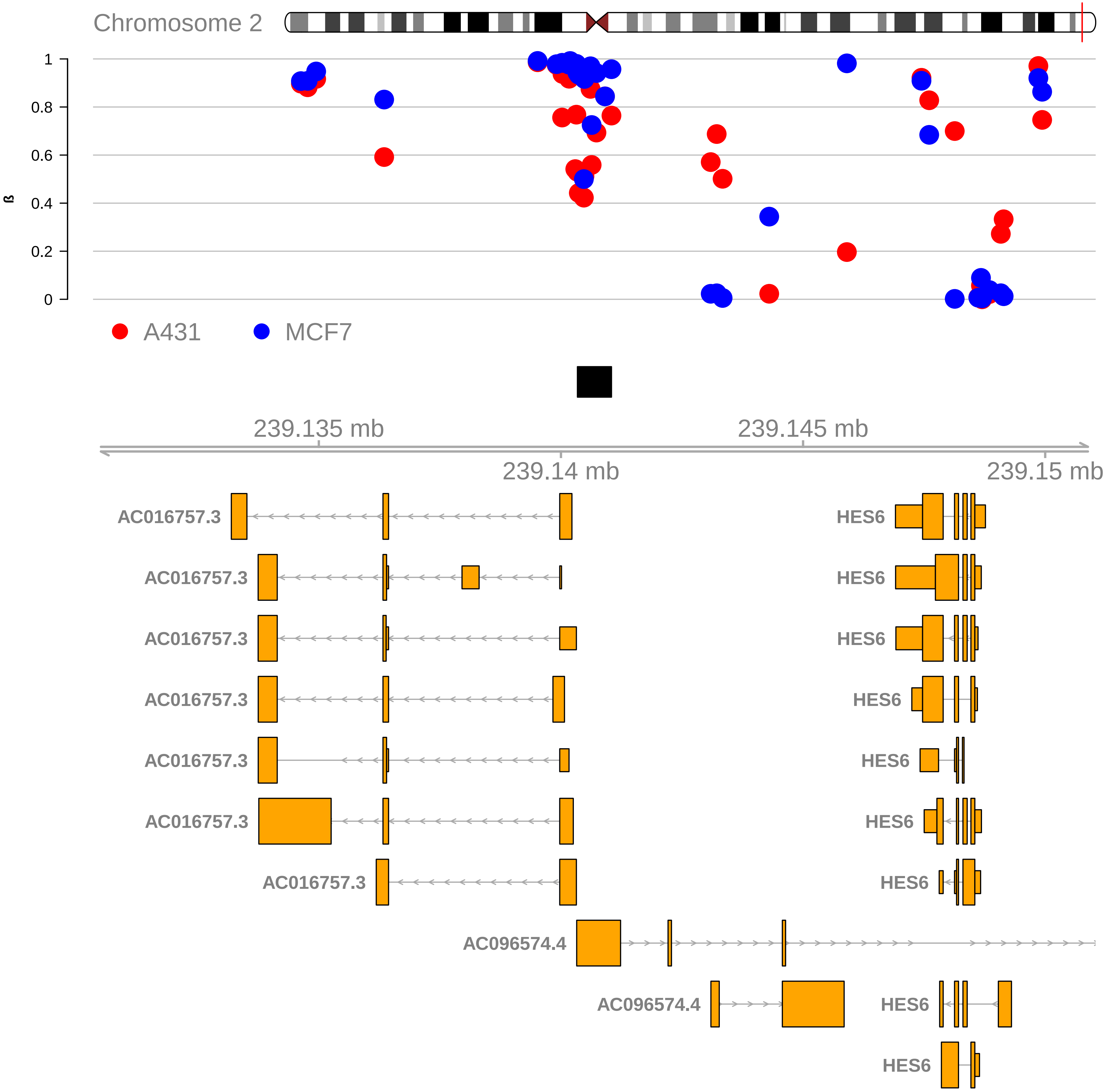

Chromosome 2
β
1
0.8
0.6
0.4
0.2
0
A431
MCF7
239.135 mb
239.145 mb
239.14 mb
239.15 mb
AC016757.3
AC016757.3
AC016757.3
AC016757.3
AC016757.3
AC016757.3
AC016757.3
AC096574.4
AC096574.4
HES6
HES6
HES6
HES6
HES6
HES6
HES6
HES6
HES6
